# Short-Term Outcomes of Colorectal Stenting Using a Low Axial Force Self-Expandable Metal Stent for Malignant Colorectal Obstruction: A Japanese Multicenter Prospective Study

**DOI:** 10.3390/jcm10214936

**Published:** 2021-10-26

**Authors:** Takashi Sasaki, Shuntaro Yoshida, Hiroyuki Isayama, Akiko Narita, Tomonori Yamada, Toshiyuki Enomoto, Yorinobu Sumida, Rika Kyo, Toshio Kuwai, Masafumi Tomita, Rintaro Moroi, Mamoru Shimada, Nobuto Hirata, Yoshihisa Saida

**Affiliations:** 1 Department of Hepato-Biliary-Pancreatic Medicine, Cancer Institute Hospital of Japanese Foundation for Cancer Research, Tokyo 135-8550, Japan; sasakit-tky@umin.ac.jp; 2 Department of Endoscopy and Endoscopic Surgery, Graduate School of Medicine, The University of Tokyo, Tokyo 113-8655, Japan; shungtang@hotmail.com; 3 Department of Gastroenterology, Graduate School of Medicine, Juntendo University, Tokyo 113-8431, Japan; 4 Department of Gastroenterology, Graduate School of Medicine, The University of Tokyo, Tokyo 113-8655, Japan; naribo_maro@yahoo.co.jp; 5 Department of Gastroenterology, Japanese Red Cross Aichi Medical Center Nagoya Daini Hospital, Aichi 466-8650, Japan; tyamada@nagoya2.jrc.or.jp; 6 Department of Surgery, Toho University Ohashi Medical Center, Tokyo 153-8515, Japan; eno@fj8.so-net.ne.jp (T.E.); yoshisaida@nifty.com (Y.S.); 7 Department of Gastroenterology, National Hospital Organization, Kyushu Medical Center and Clinical Research Center, Fukuoka 810-8563, Japan; y.raisin@gmail.com; 8 Department of Gastroenterology, Saiseikai Yokohamashi Nanbu Hospital, Kanagawa 234-0054, Japan; kyokyo134@hotmail.com; 9 Department of Gastroenterology, National Hospital Organization, Kure Medical Center and Chugoku Cancer Center, Hiroshima 737-0023, Japan; kuwai.toshio.ur@mail.hosp.go.jp; 10 Department of Surgery, Kobe Tokushukai Hospital, Hyogo 655-0017, Japan; mtomita@sc4.so-net.ne.jp; 11 Division of Gastroenterology, Department of Internal Medicine, Graduate School of Medicine, Tohoku University, Miyagi 980-8574, Japan; rinta@med.tohoku.ac.jp; 12 Department of Surgery, Toyonaka Keijinkai Hospital, Osaka 560-0004, Japan; 8.nammm@leto.eonet.ne.jp; 13 Department of Gastroenterology, Kameda Medical Center, Chiba 296-8602, Japan; nobuhira6697-kamedanonbt929@yahoo.co.jp

**Keywords:** axial force, malignant colorectal obstruction, self-expandable metal stent, safety

## Abstract

(1) Background: Endoscopic colorectal stenting with high technical success and safety is essential in discussing the oncological outcomes for the management of malignant colorectal obstruction. Mechanical properties of self-expandable metal stents are usually considered to affect clinical outcomes. (2) Methods: A multicenter, prospective study was conducted in Japan. A self-expandable metal stent with low axial force was inserted endoscopically. The primary endpoint was clinical success, defined as the resolution of symptoms and radiological findings within 24 h. Secondary endpoints were technical success and adverse events. Short-term outcomes of 7 days were evaluated in this study. (3) Results: Two hundred and five consecutive patients were enrolled. Three patients were excluded, and the remaining 202 patients were evaluated. The technical and clinical success rates were 97.5% and 96.0%, respectively. Major stent-related adverse events included stent migration (1.0%), insufficient stent expansion (0.5%), and stent occlusion (0.5%). No colonic perforation was observed. There were two fatal cases (1%) which were not related to stent placement. (4) Conclusions: The placement of self-expandable metal stents with low axial force is safe with no perforation and showed high technical and clinical success rates in short-term outcomes for the management of malignant colorectal obstruction.

## 1. Introduction

Acute colorectal obstruction causes nausea, vomiting, abdominal pain, and bowel dilation. Urgent decompressive procedures are required in this situation because it can result in bacterial translocation, electrolyte and fluid imbalance, colonic necrosis, and perforation. Malignancy is the most common cause of colorectal obstruction, and there are two causes of malignant colorectal obstructions: colorectal cancer and extra-colonic malignancies such as gastric, pancreaticobiliary, and gynecologic malignancies. The colorectal obstruction caused by advanced colorectal cancer occurs in 8–13% of cases, accounting for approximately 25% of all intestinal occlusions [1]. The most frequent cause of colorectal neoplastic obstruction is related to tumors of the sigmoid/rectal joint, splenic flexure (70%), middle rectum (10%), and anal canal (5%) [2].

Endoscopic colorectal stenting using self-expandable metal stents (SEMSs) has become widely performed for the relief of these symptoms [3]. Colorectal stent placement is widely accepted for both palliation (PAL) and as a bridge to surgery (BTS). SEMS placement as a BTS can avoid stoma creation and render oral intake possible before surgery. Colorectal stenting for PAL also maintains the patient’s quality of life. Colorectal stent placement is also performed for the obstruction caused by colorectal cancer and extra-colonic malignancies. The European Society of Gastrointestinal Endoscopy (ESGE) guidelines suggest considering colorectal stent placement as an alternative to palliative surgical decompression for extra-colonic malignancy obstruction despite the technical and clinical success rates being lower than those reported for colorectal cancer obstruction [1,2]. There are also various types of SEMSs, but it is not yet concluded which one is the best. The studies carried out so far have not shown a significant advantage for the fully covered SEMSs as compared to the uncovered SEMSs. No difference was observed in the clinical success rate between the two types of SEMSs (96% vs. 92%) and the fully covered SEMSs showed a higher migration rate (21% vs. 2%) and a trend of a lower tumor infiltration rate (4% vs. 15%) than the uncovered SEMSs [2].

Safe and successful stent placement is essential to achieve these benefits. Colonic perforation is one of the most serious complications of colorectal stent placement. Colonic perforation affects both the short-term and long-term prognosis [4]. One of the causes of perforation after SEMS placement is consistent contact between the imbedded SEMS and the colorectal mucosa. Mechanical properties of SEMSs are considered to affect the clinical outcomes of stent placement in biliary and esophageal stenting [5,6]. Axial force is the force generated when a bent SEMS is straightened. High axial force is considered a risk factor for perforation in the gastrointestinal tract [7], and thus a SEMS with a lower axial force would be expected to reduce the perforation rate.

A Niti-S Enteral Colonic Uncovered Stent, D-type (Taewoong Medical, Inc., Gimpo, Korea) is an uncovered SEMS composed of a nickel-titanium alloy (nitinol). This SEMS is a hook and cross type stent, which results in low axial force [8]. We conducted a prospective, multicenter study of colorectal stent placement using this SEMS with low axial force. In this evaluation, we focused on the short-term (7-day follow-up) safety and efficacy of this SEMS for the management of malignant colorectal obstruction.

## 2. Materials and Methods

### 2.1. Study Design

This prospective, observational, single-arm, multicenter study was conducted by the Japan Colonic Stent Safe Procedure Research Group, which received support from the Japan Gastroenterological Endoscopy Society. Before starting this prospective study, this group had developed technical guidelines for safe colonic stenting (http://colon-stent.com/, accessed on 1 August 2021) and tried to share tips with the group members for stent placement and points to avoid complications. This study was conducted at 33 institutions (8 academic centers, 25 community hospitals) of these group members. The study was approved by the institutional review board at each institution. Written informed consent to undergo the procedure and participate in this study was obtained from each patient before the procedure. This study was registered in the University Hospital Medical Information Network Clinical Trial Registry (UMIN 000011304).

### 2.2. Inclusion and Exclusion Criteria

Patients with no history of previous colorectal stenting who required treatment of malignant colorectal obstructions were enrolled in this study. A computed tomography was performed before stent placement. Malignancy of intrinsic origin was confirmed via endoscopic biopsy or macroscopic tumor findings. In cases of extrinsic tumor origin, malignancy was confirmed by computed tomography or other imaging modalities. Both indications of a BTS and PAL were included in this study. Exclusion criteria at the time of stent placement were as follows: (i) suspicion of enteral ischemia, (ii) suspected or impending perforation, (iii) the presence of intra-abdominal abscess/perforation, and (iv) any contraindication to endoscopic treatment.

### 2.3. Endoscopic Procedure

All patients received the same uncovered SEMS (Niti-S Enteral Colonic Uncovered Stent, D-type). The stent was 18 or 22 mm in diameter and 60, 80, 100, or 120 mm in length. The outer diameter of the delivery system was 10 Fr for the 22 mm diameter SEMS and 9 Fr for the 18 mm diameter SEMS. The overall length of the delivery system was 250 cm. Stent placement was performed under fluoroscopic and endoscopic guidance, in accordance with the standard procedure. A biopsy prior to stent placement was allowed. Biopsies were performed after traversing the stricture by the guidewire, because bleeding from the stricture made it difficult to identify the orifice of the stricture. To identify the stricture precisely, intraluminal or extraluminal marking using an endoscopic clip, a lipiodol, or a radiopaque marker was used at the endoscopist’s discretion. Stricture dilation before stent placement was not allowed. Although the use of CO_2_ insufflation was recommended, it was not mandatory.

### 2.4. Data Collection and Statistical Analysis

All clinical data were collected prospectively using an electronic data capture system. The data were self-reported by each investigator and the data center confirmed that there were no discrepancies. At enrolment, the treatment intent (BTS or PAL) was determined based on the stage of the malignancy, comorbidities, and, in some cases, patient choice. The extent of obstruction was classified into two groups: complete and incomplete obstruction. Complete colonic obstruction was confirmed by either of the following events: inability to pass flatus, inability of water-soluble contrast to pass proximal to the lesion, and inability to visualize the proximal lumen endoscopically [9]. The remaining cases were defined as incomplete obstruction. The ColoRectal Obstruction Scoring System (CROSS) was used to assess oral intake level and abdominal symptoms before and after the procedure: CROSS 0, requiring continuous decompression; CROSS 1, no oral intake; CROSS 2, liquid or enteral nutrient intake; CROSS 3, soft solids, low-residue, and full diet with symptoms of stricture; and CROSS 4, soft solids, low-residue, and full diet without symptoms of stricture [3].

The primary endpoint was clinical success, defined as the resolution of symptoms and radiological findings within 24 h. The secondary endpoint was technical success, defined as stent deployment across the entire length of the stricture on the first attempt. All adverse events during the study period were collected and the following conditions were considered stent-related adverse events: perforation, stent migration, insufficient stent expansion, stent occlusion, infection/fever, and abdominal pain.

Continuous variables were expressed as means and standard deviations (SD). Continuous and nominal variables were compared using the Kruskal–Wallis or χ2 test as appropriate. In this evaluation, the follow-up period was 7 days to evaluate the short-term outcomes of efficacy and safety. All analyses were performed using the JMP software (ver. 12.2.0; SAS Institute, Chicago, IL, USA).

## 3. Results

### 3.1. Baseline Characteristics

**Figure 1:**
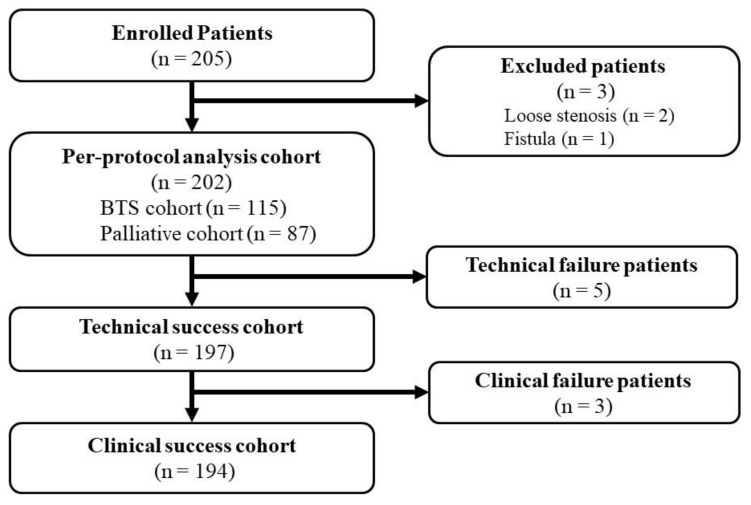
A patient flow chart of enrolled patients.

A flowchart of the patient registry is shown in Figure 1. The patients were enrolled from October 2013 to May 2014. Two hundred and five patients were enrolled in total. Three patients were then excluded because of loose stenosis identified on colonoscopy (*n* = 2) and fistula (*n* = 1). The per-protocol analysis (PPA) cohort comprised the remaining 202 patients. Treatment intent was a BTS in 115 (56.9%) patients and PAL in 87 (43.1%) patients. No patient dropped out during the 7-day follow-up period.

The patient characteristics of the PPA cohort at baseline are presented in Table 1. The mean (SD) age was 71.1 (13.9) years, and 104 (51.5%) of the patients were male. Obstructive symptoms were recorded in 193 (95.5%) patients. Regarding the CROSS classification, 69 (34.2%) patients were CROSS 0, 53 (26.2%) were CROSS 1, and 36 (17.8%) were CROSS 2. Seven (3.5%) patients with CROSS 4 had SEMSs inserted at the discretion of each doctor. Colorectal obstruction was caused by primary colorectal cancer in 173 (85.6%) patients, locally recurrent colorectal cancer in 2 (1.0%) patients, benign ulcer in 1 (0.5%) patient, and other malignant diseases in 26 (12.9%) patients. In total, 113 (55.9%) patients had localized cancer, while 88 (43.6%) patients had primary cancer with distant metastases (liver 21.8%, lung 10.9%) or peritoneal carcinomatosis (20.8%).

Tumor characteristics of the PPA cohort are presented in Table 2. Complete obstruction was present in 177 patients (87.6%). Tumor origin of extrinsic malignancy was involved in 28 (13.9%) patients. The causes of extrinsic obstruction were gastric cancers (*n* = 15), pancreatic cancers (*n* = 4), recurrent colorectal cancers (*n* = 2), bile duct cancers (*n* = 2), gynecological cancers (*n* = 2), and other cancers (*n* = 3). In total, 198 (98.0%) patients had one stricture, and 4 (2.0%) patients had two strictures. In total, 206 strictures were identified. The most common site of obstruction was the sigmoid colon (including the sigmoid-descending junction, 32.5%). The proportion of left-sided (from splenic flexure to rectum) and right-sided (from cecum to transverse colon) colorectal obstructions were 71.4% (BTS; *n* = 81, PAL; *n* = 66) and 28.6% (BTS; *n* = 34, PAL; *n* = 25), respectively.

Digestive tract decompression was performed before stent placement in 33 (16.3%) patients using a nasogastric (2.0%), nasointestinal (8.9%), or transanal (5.4%) tube. Cleansing enemas and oral bowel cleansing were performed before the procedure in 92 (45.5%) patients and 18 (8.9%) patients, respectively.

### 3.2. Clinical Outcomes of Stent Placement

Stent placement was technically successful in 197 (97.5%) patients (Table 3). As previously noted, balloon dilation of the colonic stricture was not performed before stent placement. An intraluminal stricture marker was used in 106 (52.5%) patients and an extraluminal marker was used in 22 (10.9%) patients. The mean (SD) procedure time was 36.5 (21.6) min in the technical success cohort. The mean (SD) stricture length measured during the procedure was 4.8 (2.6) cm. Eight (4.0%) patients required two SEMSs and 3 (1.5%) patients required three SEMSs. The most commonly used SEMSs were 8 cm in length (34.2%) and 22 mm in diameter (92.6%).

Technical failure was observed in five patients: inability to approach the stricture endoscopically (*n* = 1), deterioration of respiratory status before stent placement (*n* = 1), inability to release the stent (*n* = 1), inadequate stent placement (*n* = 1), and stent migration (*n* = 1). One patient with pancreatic cancer failed to pass the sigmoid colon by colonoscopy due to peritoneal carcinomatosis and to reach the stricture of the transverse colon. In the case that failed to release the Niti-S D type stent (22 mm in diameter, 10 cm in length) at a rectosigmoid stricture, a WallFlex colonic stent (22 mm in diameter, 9 cm in length) was placed instead. Inadequate stent placement occurred in one patient due to the underestimation of the stricture length.

Clinical success was achieved in 194 (96.0%) patients. Excluding the five technical failure cases, three patients did not obtain clinical success due to insufficient stent expansion (*n* = 1), stent migration (*n* = 1), and acute respiratory failure (*n* = 1). In the patient (86-year-old female) with acute respiratory failure, the general condition changed immediately after stent placement. Unfortunately, the patient died in spite of a successful stent placement; there was no finding of perforation or other complication related to the stent placement. There were no site-specific features of both technical and clinical failure in our population.

Adverse events were estimated in all the enrolled patients. There were no cases of perforation by colonoscopy insertion and the manipulation of the guidewire or catheter in this series of cases. However, there was one fatal case which discontinued the procedure due to the deterioration of the respiratory condition before stent placement. Among the PPA cohort (*n* = 202), 12 (5.9%) adverse events were observed in 11 patients (5.4%), including gastrointestinal obstruction at a proximal site in 3 (1.5%), stent migration in 2 (1.0%), and abdominal pain in 2 (1.0%). Insufficient stent expansion, stent occlusion due to fecal impaction, sepsis due to obstructive colitis, acute respiratory failure, and pneumonia were observed in one (0.5%) patient each. Although an additional SEMS was added in two cases of stent migration, the symptoms did not resolve in one case with insufficient stent expansion. A transanal tube insertion was performed in this case before surgery. Stent occlusion due to fecal impaction was managed endoscopically without further intervention. The causes of gastrointestinal obstruction at a proximal site were gastric outlet obstruction due to gastric cancer (*n* = 1) on day 5 after stenting, small intestinal obstruction due to colorectal cancer (*n* = 1) on day 6 after stenting, and obstruction of the transverse colon due to another colorectal cancer (*n* = 1) on day 5 after stenting. Gastric outlet obstruction was treated with additional gastroduodenal stent placement; the others were managed surgically. The patient (89-year-old female) with insufficient stent expansion suffered from sepsis due to obstructive colitis; emergency surgery was subsequently performed for stoma formation. Although there was no finding of perforation during surgery, she died of septic shock.

## 4. Discussion

The present study showed that colorectal stenting for malignant colorectal obstruction using a SEMS with low axial force (Niti-S Enteral Colonic Uncovered Stent, D-type) was highly effective and safe in short-term evaluation. The rates of technical success (97.5%) and clinical success (96.0%) were slightly better than those of other reports on colorectal stenting in prospective multicenter studies with technical success rates of 93–94.8% and clinical success rates of 90.5–95% [10,11]. These data were also comparable with those of our previous multicenter study using a WallFlex colonic stent with a technical success of 97.9% and a clinical success of 95.5% [12]. However, care must be taken in their interpretation since the patient backgrounds, the status of the colorectal obstruction, and the follow-up periods were different in each study. Adverse events were observed in 11 (5.4%) patients, including stent migration, insufficient stent expansion, and stent occlusion due to fecal impaction. No perforation was caused by the procedure or stent itself within 7 days after stent placement. Although two fatal cases were included in this study, they were not directly related to the stent placement. It can be explained by the fact that acute colorectal obstruction sometimes occurs in patients with extremely severe general condition. Since both patients were super elderly (86 and 89 years old), great care should be taken especially in the treatment of the super-elderly patients.

Safe and highly successful stent placement is a prerequisite for discussing the long-term outcomes of colorectal stenting. In order to obtain a high success rate, it is important to improve the skills and understand the difficult situation of colorectal stenting. Colorectal stent placement is a procedure usually performed by many types of doctors including surgeons and endoscopists who are specialized in colonoscopy or endoscopic retrograde cholangiopancreatology (ERCP). There was a report that the complications of colorectal stenting are lower when treated by a doctor who is proficient in ERCP [9]. On the contrary, it was reported that colorectal stent placement could be safely and effectively performed with short procedure time in patients with malignant colorectal obstruction after performing more than 30 procedures of endoscopic colorectal stenting [13]. We established a study group including colorectal surgeons and endoscopists who are specialized in colonoscopy and ERCP. We held a workshop to discuss and share tips on colorectal stenting and launched a website (http://colon-stent.com/, accessed on 1 August 2021) describing the standard methods of stent placement to generalize the procedure among the members. As a result, two prospective studies conducted with our study group demonstrated high technical success rates (97.5–97.9%) and clinical success rates (95.5–96.0%). It is also important to understand factors associated with technical difficulty: previous reports extracted them as complete obstruction (CROSS 0), an extra-colonic origin of the tumor, the presence of carcinomatosis, tumor site in the right colon, long stricture, and the necessity of multiple stent placements [14,15]. In such cases, it is better to perform the procedure with more experienced doctors of colorectal stenting.

Mechanical properties of SEMSs are another aspect which might affect the clinical outcomes of stent placement [16]. We previously evaluated the mechanical properties of several colorectal SEMSs [8]. The structure of the Niti-S D type is a hook and cross type, while that of a WallFlex colonic stent is a cross type. In this in vitro evaluation, the Niti-S D type stent was assigned to a group with low axial force and moderate radial force. On the other hand, the WalFlex colonic stent was assigned to a group of strong axial force and moderate radial force. We also defined a new parameter, “axial force zero border”, as the angle at which the axial force applied to the intestinal wall almost becomes zero [8]. The axial force of a SEMS gets higher when the angle of the bent SEMS is greater. As the angle of the bent SEMS gets smaller, the axial force gets lower, and it almost becomes zero at the “axial force zero border”. Therefore, when the angle of the bent SEMS is greater than the axial force zero border of the SEMS, the pressure load on the intestinal wall is sustained, which might result in perforation due to the injury of the intestinal wall. The Niti-S D type was evaluated as showing one of the largest angles of axial force zero border, which means that this SEMS places a low sustained load on the intestinal wall even when the angle of the bent SEMS is large. On the other hand, the WallFlex colonic stent was evaluated as having one of the smallest angles of axial force zero border, which means that this SEMS places a high sustained load on the intestinal wall when the angle of the bent SEMS is large. Previous prospective studies using the WallFlex colonic stent reported that the perforation rate was 2.1–3.9%, including the results of our group study (2.1%) [10,11,12]. The accumulated perforation rate of these three studies was 2.7% in total (Table 4). The present study of the Niti-S D type stent including 202 patients revealed that perforation did not occur during the short follow-up period. One meta-analysis categorized the WallFlex, ComVi stent, and Niti-S D type stent as SEMSs with higher perforation rates (>10%) compared to those of the Hanarostent and a Niti-S covered stent [17]. They concluded that the perforation rate of colorectal stenting was 7.4%, and stent design, benign etiology, and bevacizumab were identified as risk factors for perforation. We reviewed other recent publications describing colorectal stenting using the Niti-S D type stent (Table 4) [18,19,20,21,22,23,24,25,26,27,28,29]. The perforation rate of the accumulated population including the present study was 1.9%, which was slightly lower than that of the WallFlex colonic stent. There were also high differences in perforation rate observed between studies and most of the studies except the present study included a small number of patients. Moreover, some studies included procedure-related perforations [23,24,25,26]. It is necessary to distinguish between procedure-related and stent-related complications, especially when discussing the influence of the mechanical properties of SEMSs. Further evaluations of the relationship between the mechanical properties of SEMSs and clinical outcomes are needed in detail along with the accumulation of clinical data in the future.

The structure of the SEMS can also affect the technical success rate of the procedure. Since the Niti-S D type is a hook and cross type, it has a higher release resistance from the delivery system than the WallFlex colonic stent with a cross structure. Therefore, in limited cases, it may occur that the Niti-S D type cannot be deployed from the delivery system when the flexion is tight. In fact, there was one case in this study in which the Niti-S D type could not be deployed and the WallFlex colonic stent was used instead.

In the 2014 ESGE guidelines, SEMS placement is recommended as a preferred treatment for PAL cases, but it is not recommended for BTS cases as a standard treatment of symptomatic left-sided malignant colorectal obstruction [1]. Based on the accumulation of evidence, an updated version of the ESGE guidelines was published in 2020 [2]. It recommends stenting as a BTS to be discussed, within a shared decision-making process, as a treatment option in patients with potentially curable left-sided obstructing colon cancer as an alternative to emergency resection. The ESGE also suggests consideration of colorectal stenting for malignant obstruction of the proximal colon either as a BTS or PAL setting. However, long-term outcomes of colorectal stenting for malignant colorectal obstruction are still a big clinical issue. The cumulative incidence of overall recurrence in patients with perforation was significantly higher than that in patients undergoing emergency surgery or stenting without perforation [30]. The median time of stent-related perforation after stent placement is reported as 3 (range, 0–960) days in a meta-analysis [17]. Although it is also necessary to take care for delayed perforation affected by the treatment (e.g., chemotherapy and radiotherapy) after stent placement, the safe and highly successful stent placement is still considered to be the most important point.

This study includes several limitations. First, the present study was a single-arm observational study and no comparison was performed with other SEMSs such as high axial force SEMSs. However, this study prospectively evaluated a large number of patients (202 patients) compared with previous reports. Second, this study was conducted by highly experienced members who are familiar with colorectal stenting, which might affect the high technical and clinical success rate. Third, the clinical success was defined as the resolution of symptoms and radiological findings within 24 h. This judgement was performed by each investigator and patients’ symptoms were not evaluated using a questionnaire. Although it was limited, there were cases of CROSS 4 included in this study. It might be inaccurate to judge the clinical success in these patients. Finally, only short-term outcomes of colorectal stenting using a SEMS with low axial force were evaluated in this study. Since good short-term outcomes are a major premise for discussing the long-term outcomes of colorectal stenting, we have summarized the short-term outcomes in this study. The evaluation of long-term outcomes for this population is now ongoing.

In conclusion, this prospective multicenter study revealed that endoscopic colorectal stenting using a SEMS with low axial force for the management of malignant colorectal obstruction showed high short-term efficacy and safety with a low perforation rate.

## Figures and Tables

**Table 1:** Baseline patient characteristics in the per-protocol analysis of enrolled patients (*n* = 202).

Age, Years, Mean (SD)	71.1 (13.9)
Sex, % (*n*)	
Male	51.5 (104)
Female	48.5 (98)
ECOG PS, % (*n*)	
0	35.6 (72)
1	30.7 (62)
2	16.8 (34)
3	13.9 (28)
4	3.0 (6)
ASA-PS classification, % (*n*)	
1	44.6 (90)
2	39.1 (79)
3	14.9 (30)
4	1.5 (3)
Any symptoms of obstruction, % (*n*)	95.5 (193)
Deterioration of bowel habit	91.6 (185)
Bloating	80.7 (163)
Abdominal pain/cramp	75.7 (153)
Nausea or vomiting	46.0 (93)
CROSS before stent placement, % (*n*)	
0	34.2 (69)
1	26.2 (53)
2	17.8 (36)
3	18.3 (37)
4	3.5 (7)
Etiology of colorectal obstruction, % (*n*)	
Primary colorectal cancer	85.6 (173)
Locally recurrent colorectal cancer	1.0 (2)
Other extrinsic origin	12.9 (26)
Benign ulcer	0.5 (1)
Underlying disease, % (*n*)	
Only local cancer	55.9 (113)
Cancer with distant metastases	43.6 (88)
Liver metastasis	21.8 (44)
Lung metastasis	10.9 (22)
Peritoneal carcinomatosis	20.8 (42)
Other metastases	18.3 (37)
Benign lesion, % (*n*)	0.5 (1)
Treatment history, % (*n*)	
Chemotherapy	13.9 (28)
Radiotherapy	1.0 (2)
Stricture balloon dilation	0 (0)

ECOG, Eastern Cooperative Oncology Group; PS, performance status; ASA-PS, American Society of Anesthesiologists Physical Status; CROSS, ColoRectal Obstruction Scoring System.

**Table 2:** Tumor characteristics in the per-protocol analysis of enrolled patients (*n* = 202).

Complete Obstruction, % (*n*)	87.6 (177)
Tumor origin, % (*n*)	
Intrinsic malignancy	85.6 (173)
Extrinsic malignancy	13.9 (28)
Benign stricture	0.5 (1)
Single stricture, % (*n*)	98.0 (198)
Stenosis/tumor localization, * % (*n*)	
Rectum	7.3 (15/206)
Rectosigmoid junction	12.6 (26/206)
Sigmoid colon	22.8 (47/206)
Sigmoid–descending junction	9.7 (20/206)
Descending colon	12.1 (25/206)
Splenic flexure	6.8 (14/206)
Transverse colon	12.1 (25/206)
Hepatic flexure	5.3 (11/206)
Ascending colon	7.3 (15/206)
Cecum	3.9 (8/206)
Ileocecal junction	0 (0/206)

* Total number of the stricture: 206 in total because two different strictures were identified in four cases.

**Table 3:** Clinical outcomes of stent placement.

Technical success, % (*n*)	97.5 (197/202)
Technical failure, % (*n*)	
Inability to approach the stricture endoscopically	0.5 (1/202)
Deterioration of respiratory status before stent placement	0.5 (1/202)
Inability to release the stent	0.5 (1/202)
Inadequate stent placement	0.5 (1/202)
Stent migration	0.5 (1/202)
Clinical success, % (*n*)	96.0 (194/202)
Clinical failure, % (*n*)	
Patients with technical failure	2.5 (5/202)
Insufficient stent expansion	0.5 (1/202)
Stent migration	0.5 (1/202)
Acute respiratory failure	0.5 (1/202)
Procedure time in the technical success cohort, min., mean (SD)	36.5 (21.6)
Stricture length, cm, mean (SD)	4.8 (2.6)
Strictures and stents placed, % (*n*)	
Double stricture with no stent (technical failure)	0.5 (1/202)
Single stricture with 1 stent	93.6 (189/202)
Single stricture with 2 stents	3.0 (6/202)
Single stricture with 3 stents	1.0 (2/202)
Double stricture with 2 stents	1.0 (2/202)
Double stricture with 3 stents	0.5 (1/202)
Stent type, % (*n*)	
Niti-S Enteral Colonic Uncovered Stent, D type	99.5 (213/214)
18 mm in diameter	6.1 (13/214)
22 mm in diameter	93.4 (200/214)
6 cm in length	20.1 (43/214)
8 cm in length	34.6 (74/214)
10 cm in length	28.0 (60/214)
12 cm in length	16.8 (36/214)
WallFlex colonic stent	0.5 (1/214)
22 mm in diameter	0.5 (1/214)
9 cm in length	0.5 (1/214)

**Table 4:** Previous studies for colorectal stenting using Niti-S D type and WallFlex colonic stents.

Study	Year	Country	Design	Patients	Perforation	Perforation Rate
Niti-S D type						
Lee, K.M. [18]	2007	Korea	Prospective	59	0	0%
Shin, S.J. [19]	2008	Korea	Retrospective	38	0	0%
Pommergaard, H.C. [20]	2009	Denmark	Retrospective	3	1	33.3%
Reza, F. [21]	2009	Iran	Prospective	8	0	0%
Kim, J.S. [22]	2009	Korea	Retrospective	18	0	0%
Moon, C.M. [23]	2010	Korea	Prospective	37	1	2.7%
Jung, M.K. [24]	2010	Korea	Retrospective	17	2	11.8%
Lee, H.J. [25]	2011	Korea	Retrospective	17	3 *	17.6%
Park, J.K. [26]	2011	Korea	Retrospective	20	0	0%
Iverson, L.H. [27]	2011	Denmark	Retrospective	4	2	50.0%
Tominaga, K. [28]	2012	Japan	Prospective	24	0	0%
Yoshida, S. [29]	2013	Japan	Prospective	33	0	0%
Present study	2021	Japan	Prospective	202	0	0%
Total				480	9	1.9%
WallFlex colonic stent						
Repici, A. [10]	2008	Italy	Prospective	42	1	2.4%
Meisner, S. [11]	2011	Global	Prospective	447	15	3.4%
Matsuzawa, T. [12]	2015	Japan	Prospective	513	11	2.1%
Total				1002	27	2.7%

* Because there is no description about the cases with perforation in the article, we calculated the number from the systematic review [17].

## Data Availability

Data sharing is not applicable.

## References

[1] van Hooft J.E., van Halsema E.E., Vanbiervliet G., Beets-Tan R.G., DeWitt J.M., Donnellan F., Dumonceau J.M., Glynne-Jones R.G., Hassan C., Jiménez-Perez J. (2014). Self-expandable metal stents for obstructing colonic and extracolonic cancer: European Society of Gastrointestinal Endoscopy (ESGE) Clinical Guideline. Endoscopy.

[2] van Hooft J.E., Veld J.V., Arnold D., Beets-Tan R.G.H., Everett S., Götz M., van Halsema E.E., Hill J., Manes G., Meisner S. (2020). Self-expandable metal stents for obstructing colonic and extracolonic cancer: European Society of Gastrointestinal Endoscopy (ESGE) Guideline—Update 2020. Endoscopy.

[3] Saida Y. (2019). Current status of colonic stent for obstructive colorectal cancer in Japan; a review of the literature. J. Anus Rectum Colon..

[4] Balciscueta I., Balciscueta Z., Uribe N., García-Granero E. (2020). Long-term outcomes of stent-related perforation in malignant colon obstruction: A systematic review and meta-analysis. Int. J. Colorectal Dis..

[5] Isayama H., Nakai Y., Toyokawa Y., Togawa O., Gon C., Ito Y., Yashima Y., Yagioka H., Kogure H., Sasaki T. (2009). Measurement of radial and axial forces of biliary self-expandable metallic stents. Gastrointest. Endosc..

[6] Hirdes M.M., Vleggaar F.P., de Beule M., Siersema P.D. (2013). In vitro evaluation of the radial and axial force of self-expanding esophageal stents. Endoscopy.

[7] Sasaki T., Isayama H., Nakai Y., Takahara N., Hamada T., Mizuno S., Mohri D., Yagioka H., Kogure H., Arizumi T. (2015). Clinical outcomes of secondary gastroduodenal self-expandable metallic stent placement by stent-in-stent technique for malignant gastric outlet obstruction. Dig. Endosc..

[8] Sasaki T., Ishibashi R., Yoshida S., Fujisawa T., Shinagawa H., Gon C., Nakai Y., Sasahira N., Saida Y., Isayama H. (2021). Comparing the mechanical properties of a self-expandable metallic stent for colorectal obstruction: Proposed measurement method of axial force using a new measurement machine. Dig. Endosc..

[9] Small A.J., Coelho-Prabhu N., Baron T.H. (2010). Endoscopic placement of self-expandable metal stents for malignant colonic obstruction: Long-term outcomes and complication factors. Gastrointest. Endosc..

[10] Repici A., De Caro G., Luigiano C., Fabbri C., Pagano N., Preatoni P., Danese S., Fuccio L., Consolo P., Malesci A. (2008). WallFlex colonic stent placement for management of malignant colonic obstruction: A prospective study at two centers. Gastrointest. Endosc..

[11] Meisner S., González-Huix F., Vandervoort J.G., Goldberg P., Casellas J.A., Roncero O., Grund K.E., Alvarez A., García-Cano J., Vázquez-Astray E. (2011). Self-expandable metal stents for relieving malignant colorectal obstruction: Short-term safety and efficacy within 30 days of stent procedure in 447 patients. Gastrointest. Endosc..

[12] Matsuzawa T., Ishida H., Yoshida S., Isayama H., Kuwai T., Maetani I., Shimada M., Yamada T., Saito S., Tomita M. (2015). A Japanese prospective multicenter study of self-expandable metal stent placement for malignant colorectal obstruction: Short-term safety and efficacy within 7 days of stent procedure in 513 cases. Gastrointest. Endosc..

[13] Lee H.J., Park S.J., Cheon J.H., Kim T.I., Kim W.H., Hong S.P. (2015). What is the necessity of endoscopist for successful endoscopic stenting in patients with malignant colorectal obstruction?. Int. J. Color. Dis..

[14] Yoon J.Y., Jung Y.S., Hong S.P., Kim T.I., Kim W.H., Cheon J.H. (2011). Clinical outcomes and risk factors for technical and clinical failures of self-expandable metal stent insertion for malignant colorectal obstruction. Gastrointest. Endosc..

[15] Kuwai T., Yamaguchi T., Imagawa H., Yoshida S., Isayama H., Matsuzawa T., Yamada T., Saito S., Shimada M., Hirata N. (2019). Factors related to difficult self-expandable metallic stent placement for malignant colonic obstruction: A post-hoc analysis of a multicenter study across Japan. Dig. Endosc..

[16] Isayama H., Nakai Y., Kogure H., Hamada T., Yamamoto N., Koike K. (2014). Can we develop self-expandable metallic stents without consideration of mechanical properties?. Endoscopy.

[17] van Halsema E.E., van Hooft J.E., Small A.J., Baron T.H., García-Cano J., Cheon J.H., Lee M.S., Kwon S.H., Mucci-Hennekinne S., Fockens P. (2014). Perforation in colorectal stenting: A meta-analysis and a search for risk factors. Gastrointest. Endosc..

[18] Lee K.M., Shin S.J., Hwang J.C., Cheong J.Y., Yoo B.M., Lee K.J., Hahm K.B., Kim J.H., Cho S.W. (2007). Comparison of uncovered stent with covered stent for treatment of malignant colorectal obstruction. Gastrointest. Endosc..

[19] Shin S.J., Kim T.I., Kim B.C., Lee Y.C., Song S.Y., Kim W.H. (2008). Clinical application of self-expandable metallic stent for treatment of colorectal obstruction caused by extrinsic invasive tumors. Dis. Colon Rectum..

[20] Pommergaard H.C., Vilmann P., Jakobsen H.L., Achiam M.P. (2009). A clinical evaluation of endoscopically placed self-expanding metallic stents in patients with acute large bowel obstruction. Scand. J. Surg..

[21] Reza F., Amir M.A., Faramarz D., Shahrokh M., Mehrdad Z., Shivarani S., Malek F.N., Maserat E., Zali M.R. (2009). Colorectal stenting for management of acute malignant bowel obstruction in advanced colorectal cancer in Iran. Asian Pac. J. Cancer Prev..

[22] Kim J.S., Hur H., Min B.S., Sohn S.K., Cho C.H., Kim N.K. (2009). Oncologic outcomes of self-expanding metallic stent insertion as a bridge to surgery in the management of left-sided colon cancer obstruction: Comparison with nonobstructing elective surgery. World J. Surg..

[23] Moon C.M., Kim T.I., Lee M.S., Ko B.M., Kim H.S., Lee K.M., Byeon J.S., Kim Y.S. (2010). Comparison of a newly designed double-layered combination covered stent and D-weave uncovered stent for decompression of obstructive colorectal cancer: A prospective multicenter study. Dis. Colon Rectum..

[24] Jung M.K., Park S.Y., Jeon S.W., Cho C.M., Tak W.Y., Kweon Y.O., Kim S.K., Choi Y.H., Kim G.C., Ryeom H.K. (2010). Factors associated with the long-term outcome of a self-expandable colon stent used for palliation of malignant colorectal obstruction. Surg. Endosc..

[25] Lee H.J., Hong S.P., Cheon J.H., Kim T.I., Min B.S., Kim N.K., Kim W.H. (2011). Long-term outcome of palliative therapy for malignant colorectal obstruction in patients with unresectable metastatic colorectal cancers: Endoscopic stenting versus surgery. Gastrointest. Endosc..

[26] Park J.K., Lee M.S., Ko B.M., Kim H.K., Kim Y.J., Choi H.J., Hong S.J., Ryu C.B., Moon J.H., Kim J.O. (2011). Outcome of palliative self-expanding metal stent placement in malignant colorectal obstruction according to stent type and manufacturer. Surg. Endosc..

[27] Iversen L.H., Kratmann M., Bøje M., Laurberg S. (2011). Self-expanding metallic stents as bridge to surgery in obstructing colorectal cancer. Br. J. Surg..

[28] Tominaga K., Maetani I., Sato K., Shigoka H., Omuta S., Ito S., Saigusa Y. (2012). Favorable long-term clinical outcome of uncovered D-weave stent placement as definitive palliative treatment for malignant colorectal obstruction. Dis. Colon Rectum..

[29] Yoshida S., Watabe H., Isayama H., Kogure H., Nakai Y., Yamamoto N., Sasaki T., Kawakubo K., Hamada T., Ito Y. (2013). Feasibility of a new self-expandable metallic stent for patients with malignant colorectal obstruction. Dig. Endosc..

[30] Sloothaak D.A., van den Berg M.W., Dijkgraaf M.G., Fockens P., Tanis P.J., van Hooft J.E., Bemelman W.A., collaborative Dutch Stent-In study group (2014). Oncological outcome of malignant colonic obstruction in the Dutch Stent-In 2 trial. Br. J. Surg..

